# Supplementary material for: Prevalence of Vitamin D Deficiency in Treatment-Naïve Subjects with Chronic Pulmonary Aspergillosis
Source: J Fungi (Basel). 2020 Oct 1;6(4):202. doi: 10.3390/jof6040202 (PMC7712853; doi:10.3390/jof6040202)
Supplement: Supplementary file 1 [file jof-06-00202-s001.pdf]

Journal of Fungi

MDPI

Supplementary Materials: Prevalence of Vitamin D Deficiency in Treatment-Naïve Subjects with Chronic Pulmonary Aspergillosis

Table S1. Diagnostic criteria for diagnosis of chronic pulmonary aspergillosis.

**Presence of an Underlying Structural Lung Disease**
• Post-pulmonary tuberculosis sequalae, interstitial lung disease, allergic bronchopulmonary aspergillosis, chronic obstructive pulmonary disease, bronchiectasis, post-thoracic surgery (lobectomy, pneumonectomy and others), and others
**Clinical Presentation**
• Duration of symptoms (at least 3 months)<br>• Symptoms: systemic (fever, weight loss, anorexia, malaise, fatigue), respiratory (cough, expectoration, dyspnea, chest pain, hemoptysis)
**Evidence of *Aspergillus* Infection**
• Respiratory tract cultures showing *Aspergillus* species on more than one occasion<br>• *A.fumigatus*-specific IgG >27 mgA/L (using the ImmunoCap method)*<br>• Demonstration of *Aspergillus* in respiratory secretions by molecular methods<br>• Elevated serum (>0.6) or bronchoalveolar lavage fluid (>1.6) galactomannan*
**Radiological Manifestations**
• One or more cavities, pulmonary fibrosis, fungal ball, pleural thickening, and nodules<br>• Simple aspergilloma: single thin walled cavity with a fungal ball without any pleural thickening or infiltrates surrounding the cavity<br>• Chronic cavitary pulmonary aspergillosis (CCPA): presence of one or more cavities with or without fungal ball. Most patients have thick walled cavities with pericavitary infiltrates including nodules, consolidation and parenchymal fibrosis<br>• Chronic fibrosing pulmonary aspergillosis: destruction of at least two lobes of lung due to fibrosis. Destruction of one lobe is classified as CCPA
**Exclusion of other Causes**
• Exclusion of pulmonary disorders with similar presentation such as tuberculous and non-tuberculous mycobacterial infection, chronic necrotizing pneumonias, pulmonary malignancies, and others

* The cut-off value may be different in different geographical locale, method used for estimation, and the study population and needs to be derived.
